# Predictors of Psychological Distress in Health Care Staff in Qatar during COVID-19 Pandemic

**DOI:** 10.12669/pjms.37.7.4533

**Published:** 2021

**Authors:** Finza Latif, Sawssan R Ahmed, Sumera Farhan, Felice Watt, Muhammad Waqar Azeem

**Affiliations:** 1 Finza Latif, MD, DFAACAP. Assistant Professor, Department of Psychiatry, Sidra Medicine, Weill Cornell Medicine-Qatar, Doha, Qatar; 2 Sawssan R. Ahmed, PhD. Clinical Psychologist, Department of Psychiatry, Sidra Medicine, Doha, Qatar; 3 Sumera Farhan, MBA. Occupational Health Specialist, Department of Occupational Health, Sidra Medicine, Doha, Qatar; 4 Felice Watt, MBBS. Assistant Professor, Department of Psychiatry, Sidra Medicine, Weill Cornell Medicine-Qatar, Doha, Qatar; 5 Muhammad Waqar Azeem, MD, DFAACAP, DFAPA. Professor of Psychiatry, Department of Psychiatry, Sidra Medicine, Weill Cornell Medicine-Qatar, Doha, Qatar

**Keywords:** Mental health, Social isolation, Expatriates, Pandemic

## Abstract

**Background & Objective:**

The Coronavirus disease 2019 (COVID-19) pandemic has caused widespread psychological distress. The aims of the study were a) to assess mental health symptoms experienced by expatriate hospital staff and b) to determine the impact of staff wellbeing interventions specific to pandemic related stress.

**Methods:**

The study was conducted from June 2020 until August 2020. A 16-question survey was disseminated online via Survey Monkey to assess the mental health needs of hospital staff during the pandemic. Based on results, a virtual, tiered mental health support model was developed, and staff feedback was collected.

**Results:**

Almost 46.2% of respondents (N: 1001) reported at least one mental health symptom in the initial survey. The most common symptoms were anxiety, low mood and feelings of isolation. Being single and in poor health status were predictors of developing mental health symptoms (P <0.01). Female gender was a predictor for experiencing fear of getting infected. Time constraints at work was the most common reason for not accessing mental health support.

**Conclusions:**

As in other parts of the world, hospital staff in Qatar experienced mental health symptoms and significant fear related to the COVID-19 Pandemic. Being single and in poor health status were risk factors. Mental health interventions at work must take into account time constraints experienced by staff.

## INTRODUCTION

The WHO declared Coronavirus disease 2019 (COVID-19) a global pandemic on March 11^th^, 2020. The first case of the virus in the small gulf state of Qatar was reported on February 28^th^, 2020. As the rate of infections grew, the State of Qatar issued gradually tightening social restrictions starting early March, 2020.^1^ The restrictions included closures of schools, shopping malls, parks and recreation venues and travel out of the country. In April, 2020 the Ministry of Public Health reported 106,648 cases of Coronavirus Disease (COVID-19) in Qatar, which has a population of 2.7 million. Total number of deaths was 157.^2^

Health care staff are at increased risk for experiencing adverse mental health symptoms during a pandemic.^3^ Previous studies^4^,^5^ have shown that during a pandemic hospital staff had concerns about their personal safety, about transmitting the disease to family members, stigmatization and interpersonal isolation. Studies have shown that hospital staff who felt their work environment was supportive^5^ and those that had access to intense psychiatry liaison services^6^ reported less stress and more motivation at work. Proactively providing mental health support to hospital staff can prevent future mental health difficulties in this population, decreasing the likelihood of burnout and loss of workforce.^7^

At the onset of the pandemic, the study hospital was designated as COVID-free, as in any confirmed cases of COVID-19 were to receive medical care in a different hospital system. Despite not having a COVID specific unit, hospital managers and staff reported psychological distress anecdotally. More than 85% of Qatar’s population comprises of expatriates, similarly the majority of the hospital staff were residents of other countries. Pandemic related social restrictions increase feelings of loneliness and the risk for depression, anxiety and insomnia.^8^ Likely Travel restrictions led to higher risk of loneliness for our study population due to their expatriate status and distance from their extended families.

The objectives of this study were (a) to assess mental health symptoms experienced by expatriate hospital staff and (b) to determine the impact of staff wellbeing interventions specific to pandemic related stress.

## METHODS

The study site was a 300 bed women and children’s hospital and research center, which employs 4500 staff from over 90 countries worldwide. A taskforce to study the impact of the pandemic and develop staff wellbeing interventions was created by the Departments of Occupational Health and Psychiatry. Stakeholder buy in was obtained via collaboration with the hospital Covid-19 Outbreak Prevention and Control Taskforce (OPCT). Staff Mental Health was added as a priority to the OPCT agenda.

### Ethical Approval:

The study was approved by the Sidra Medicine Institutional Review Board. (Ref: 1617674, Dated: June 28, 2020).

A literature review of the psychological impact on health care workers during past pandemics was conducted to determine common mental health symptoms experienced by healthcare staff. This information was used to develop a survey that included questions specific to pandemic related stressors e.g. loss of family members due to COVID-19. In addition, the survey asked about staff interest in wellbeing interventions. Thus the survey served a dual goal of assessing psychological distress level and a needs assessment prior to implementing mental health interventions for health care staff. The survey was reviewed by content experts. It was also piloted within the content expert group for accuracy of content and ease of use.

The survey was sent out in June 2020. This survey included questions regarding participant’s age, gender, profession, nationality, marital status and perceived health status. Additionally, participants were asked about personal or family exposure to COVID-19 and death of a family member or close friend due to COVID-19. The survey asked participants to answer yes if they experienced the following mental health symptoms: anxiety, irritability/anger, insomnia, low mood, poor concentration, indecisiveness/difficulty making decisions, loss of motivation, avoidance of people or situations, feelings of isolation or none. The survey also asked the participants to rate their fear of contracting the virus and fear of infecting others on a continuous variable scale from 0-100. (See supplementary material for full survey). Finally, the survey asked about staff interest in attending mental health webinars, virtual support groups and individual counseling for psychological symptoms.

### Survey administration

The survey was administered online on Survey Monkey in May 2020, two months following the initial shut down. It was further advertised via email to managers, Division Chiefs and Department Chairs. Additionally, banners were uploaded to the hospital web-portal. It was available for completion for a period of two weeks. The results were collected anonymously.

**Fig.1:**
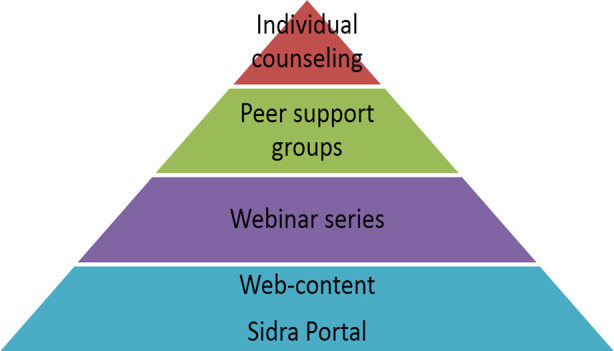
Tiered Mental Health Support model.

Following the survey, a tiered approach using online tools to deliver mental health support within the hospital was developed. The tiers included non-personalized curated web-content, mental health webinars offered biweekly for 8 weeks (June-July 2020), smaller virtual peer support groups and individual counseling (Fig.1). The webinars and virtual peer support groups were advertised on the hospital web-portal.

### Data Analysis

A composite variable was created to analyze mental health symptoms, which aggregated the nine self-reported mental health symptoms listed in Table-II. If a respondent answered yes, that they experienced a symptom, then that symptom was scored as 1 and if they answered no then it was scored as 0, for a total possible range of scores for mental health symptoms of 0-9. Multiple regression analyses were conducted to test if age, gender, marital status, profession and self-reported health status predicted number of mental health symptoms. Multiple regression analyses were also conducted to test if age, gender, marital status, profession and self-reported health status predicted whether a respondent reported they had a fear of infecting others or fear of becoming infected.

## RESULTS

### Survey Response rates

The initial survey was completed by 1001 hospital staff which was 20% of all staff. Response rate was 18% (N:130/702) for physicians, 36% (N:455/1262) for nursing and 21.7% (N:187/860) for Allied Health. The remaining staff were in non-patient facing services (N=222). A few participants skipped the question about their staff role (N:7).

### Demographics

Only 3.9% of the participants were Qatari Nationals. The majority of participants were from the Philippines and the United Kingdom, followed by India, Jordan, Ireland and the United States. The majority of staff were female (71.2%; N:715), reported they were in good health (N: 775; 77%), married (N: 638; 63.4 %) and between the ages of 25-34 years (N: 472; 46.8%) (Table-I).

**Table I:** Demographics and Health Status. (Total N=1001).

		*Frequency*	*Valid Percent %*
Specialty	Medical	130	13.08 45.77 18.01 18.11 5.03
	Nursing	455	
	Allied health	179	
	Non Clinical	180	
	Other	50	
	Missing	7	
Gender	Male	283	28.53 71.47
	Female	709	
	Missing	9	
Age groups	18-24	10	1.00 46.89 29.72 15.46 6.43 0.50
	25-34	467	
	35-44	296	
	45-54	154	
	55-64	64	
	65+	5	
	Missing	5	
Nationality	Australia	19	1.95 3.18 1.02 11.59 3.90 4.31 2.46 1.13 1.64 29.13 35.59 1.44 3.90 16.82 4.82 9.12
	Canada	31	
	Egypt	10	
	India	113	
	Ireland	38	
	Jordan	42	
	Lebanon	24	
	New Zealand	11	
	Pakistan	16	
	Philippines	284	
	South Africa	35	
	Sudan	14	
	Qatar	38	
	United Kingdom	164	
	United States	47	
	Other	89	
	Missing	26	
Marital Status	Married	630	63.32 36.68
	Single	365	
	Missing	6	
Health status	Good/Fair	766	77.14 18.23 3.93 0.70
	Minor health problems	181	
	Moderate	39	
	Severe health problems	7	
	Missing	8	

### Mental health symptoms

46.2% (N: 463) of respondents reported at least one mental health symptom. 46% of staff reported anxiety (N: 462), 41% reported low mood (N: 419) and 36.8% reported feelings of isolation (N:370). (Table-II). The mean score for the mental health composite was 3.0 (SD=2.3).

**Table II:** Survey Responses.

*Question*	*Response*	*Frequency*	*Valid Percent (%)*
Family member contracted COVID-19	Yes	38	3.82
	No	956	96.18
	Missing	7	
Death of Family member or close friend due to COVID-19	Yes	42	4.20
	No	958	95.80
	Missing	1	
Mandatory Quarantine	Yes	133	13.31
	No	865	86.59
	Missing	2	
Direct involvement in COVID-19 patient care	Yes	422	42.33
	No	575	57.67
	Missing	4	
Mental Health Concerns	Anxiety	457	47.06
	Low mood	413	42.53
	Insomnia	351	36.15
	Irritability/anger	245	25.23
	Poor concentration	216	22.25
	Indecisiveness / difficulty making decisions	115	11.84
	Loss of motivation	356	36.66
	Avoidance of people or situations	327	33.68
	Feelings of isolation	365	37.59
	None	174	17.92
	Missing	30	

Regression analysis revealed that marital status predicted number of mental health symptoms with those with a single marital status reporting more mental health symptoms (β=-.14, *p<*.001) than their married colleagues. Additionally, health status predicted mental health symptoms in the expected direction with staff who reported worse health status reporting more mental health symptoms (β=.17, *p<*.001). No other significant predictors of mental health symptoms were found.

Fear of getting infected at work and infecting others: The mean fear rating for getting infected at work was 62.3 (SD 29.3, min=0, max=100) and fear of infecting others was 61.9 (SD 32.4; min=0, max=100).

Gender (β=.12, *p<*0.001), age (β=-0.18, *p<*0.001) and marital status (β=-0.10, *p<*0.01) and health status ((β=0.16, *p<*0.001) predicted fear of being infected at work. Participants who were female, of younger age, married and who reported poorer health were more likely to have a greater fear of being infected at work.

Gender (β=.10, *p<*.01), age (β=-.27, *p<*.001) health status ((β=.11, *p<*.001) and profession (β=-.09, *p<*.01) predicted fear of infecting others. Participants who were female, of younger age, worked in a non-clinical role and who reported poorer health were more likely to have a greater fear of infecting others.

Level of interest in wellbeing interventions and mean attendance: (scale 0-100) The mean level of interest expressed by staff for general webinars was 52.7 (SD=33.0), virtual support groups was 47.8 (SD=32.6) and individual counseling was 51.1 (SD=34.3) in the initial survey. The mean number of participants that attended webinars was 77.6 (SD=30.2, min=31, max=122) and 0 for both virtual support groups and individual counseling.

**Fig.2:**
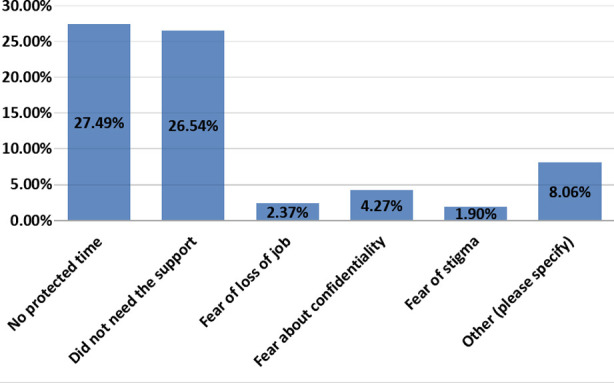
Staff Feedback on attendance.

The two most common reasons reported for non-attendance were ‘No protected time’ and ‘Felt I did not need it’ (Fig.2).

## DISCUSSION

Hospital staff reported high levels of anxiety, low mood and feelings of isolation. Staff also reported significant fear of infecting themselves at work and infecting others. These findings are consistent with previous findings related to healthcare staff during other viral pandemics e.g., SARS-COV-1,^4^,^8^,^11^ H1N1,^6^,^12^ MERS-cov^5^,^13^,^15^ and Ebola^16^ and during the COVID-19 Pandemic in other countries.^17^,^18^ Female gender was associated with higher fear ratings but there was no difference in reports of mental health symptoms based on gender unlike findings in other studies where female gender was at higher risk.^18^

Social isolation is a well-known risk factor for poor psychological and physical health.^19^,^20^ In this study, feeling of isolation was the third most commonly experienced symptom and single staff reported higher rates of mental health symptoms compared to married staff, supporting the view that social support is a protective factor for mental health.^21^ Given that more than 85% of the hospital staff was comprised of expatriates with family members in various countries around the world and travel restrictions meant that staff no longer had access to extended families in case of personal or family sickness, being single likely posed a bigger challenge than elsewhere in the world.

Hospital staff reported significant fear of infecting themselves or their loved ones which is a significant contributor to psychological distress compared to the general population, as shown in several studies.^9^,^18^,^22^ Married staff reported higher fear of getting infected at work. Therefore, although being married was protective against mental health symptoms, it was associated with higher fears and concerns related to the health of loved ones. Other studies^23^ have reported distress secondary to health care staff concerns endangering their families if they were to become infected at work. Staff in non-clinical roles were more likely to report fear of infecting others. Greater knowledge of and experience with infection control and prevention methods may have served as a protective factor against fear of infection for clinical staff.^24^

Despite high levels of mental health symptoms and fear reported by hospital staff, and similar to results in China,^25^ staff did not access the personalized peer support groups or individual counseling. In comparison, the webinars which were recorded and available online had consistently high attendance every week. Staff cited time constraints and lack of perceived need as the reasons for lack of attendance in virtual support groups or individual counseling. The webinars did not mandate consistent attendance and were recorded and available at the staff member’s convenience thus overcoming the issue of time constraints during work hours. The non-personalized, educational webinars were probably also more acceptable compared to the smaller, per support groups due to lower associated stigma. Additionally, the webinars offered a means for social connectedness within the hospital community thus reducing the sense of isolation.

### Limitations of the study

Limitations include a low response rate with the majority of respondents being nurses. Therefore, it may not adequately capture the mental health impact on non-nursing health care staff.

Further, response bias may exist, possibly missing staff who were too distressed to respond or not distressed and therefore not interested in responding. The survey does not differentiate pre-existing psychological difficulties from new pandemic related psychological distress. Finally, the study does not report on longitudinal progression of mental health impact as the pandemic progressed and after mental health interventions were implemented. It must be emphasized that this study was conducted in a non-COVID hospital which may have impacted level of psychological distress findings.

## CONCLUSION

This study provides information on psychological symptoms experienced by health care staff during the viral pandemic in a socially restricted environment. As expected, respondents reported moderate levels of mental health symptoms. Single status was associated with higher psychological distress, likely related to social isolation caused by travel and social distancing restrictions and not having a spouse or partner during this isolation.

Dissemination of mental health support in the form of webinars offering viewing flexibility was well received by staff while more personalized and time consuming approaches like peer support groups and individualized counseling were not utilized at all. Lack of time was the most commonly cited as reason. This is an important finding for planning future wellbeing initiatives for busy health care staff as the COVID-19 Pandemic progresses.

### Author’s Contribution:

**FL, SA & FW:** Contributed to the design of the study, Data collection, Review of analysis and writing of the manuscript. These authors are responsible for the accuracy and integrity of the work presented.

**SF:** Conducted the analysis for the study.

**WA:** Contributed to the writing of the manuscript.
